# Fatty infiltration in the musculoskeletal system: pathological mechanisms and clinical implications

**DOI:** 10.3389/fendo.2024.1406046

**Published:** 2024-06-28

**Authors:** Yihua Zhu, Yue Hu, Yalan Pan, Muzhe Li, Yuanyuan Niu, Tianchi Zhang, Haitao Sun, Shijie Zhou, Mengmin Liu, Yili Zhang, Chengjie Wu, Yong Ma, Yang Guo, Lining Wang

**Affiliations:** ^1^ Laboratory of New Techniques of Restoration & Reconstruction, Institute of Traumatology & Orthopedics, Nanjing University of Chinese Medicine, Nanjing, Jiangsu, China; ^2^ School of Integrated Chinese and Western Medicine, Nanjing University of Chinese Medicine, Nanjing, Jiangsu, China; ^3^ Traditional Chinese Medicine (TCM) Nursing Intervention Laboratory of Chronic Disease Key Laboratory, Nanjing University of Chinese Medicine, Nanjing, Jiangsu, China; ^4^ Department of Orthopedic Surgery, Affiliated Huishan Hospital of Xinglin College of Nantong University, Wuxi, Jiangsu, China; ^5^ Yancheng TCM Hospital Affiliated to Nanjing University of Chinese Medicine, Yancheng TCM Hospital, Yancheng, Jiangsu, China; ^6^ Jiangsu CM Clinical Innovation Center of Degenerative Bone & Joint Disease, Wuxi TCM Hospital Affiliated to Nanjing University of Chinese Medicine, Wuxi, Jiangsu, China; ^7^ Chinese Medicine Centre (International Collaboration between Western Sydney University and Beijing University of Chinese Medicine), Western Sydney University, Sydney, Australia

**Keywords:** aging, fatty infiltration, lipotoxicity, musculoskeletal system, adipose tissue

## Abstract

Fatty infiltration denotes the anomalous accrual of adipocytes in non-adipose tissue, thereby generating toxic substances with the capacity to impede the ordinary physiological functions of various organs. With aging, the musculoskeletal system undergoes pronounced degenerative alterations, prompting heightened scrutiny regarding the contributory role of fatty infiltration in its pathophysiology. Several studies have demonstrated that fatty infiltration affects the normal metabolism of the musculoskeletal system, leading to substantial tissue damage. Nevertheless, a definitive and universally accepted generalization concerning the comprehensive effects of fatty infiltration on the musculoskeletal system remains elusive. As a result, this review summarizes the characteristics of different types of adipose tissue, the pathological mechanisms associated with fatty infiltration in bone, muscle, and the entirety of the musculoskeletal system, examines relevant clinical diseases, and explores potential therapeutic modalities. This review is intended to give researchers a better understanding of fatty infiltration and to contribute new ideas to the prevention and treatment of clinical musculoskeletal diseases.

## 1 Introduction

Musculoskeletal degenerative diseases such as osteoporosis, sarcopenia, and osteosarcopenia are becoming more common as society ages, and the damage to the system not only affects patients’ quality of life but also imposes a serious socioeconomic burden (1). The musculoskeletal system is a motion regulation system made up of bones, muscles, ligaments, tendons, and articular cartilage that is in charge of maintaining normal body activities as well as certain endocrine and immune functions (2). The skeleton and muscle, as the main structures of the system, all belong to mesodermal cells, both are mechanically reactive tissues whose mass and strength show significant degenerative changes with aging or other pathological factors causing abnormal accumulation of adipocytes. The ascending and descending correspondence between adipose, bone, and muscle is the starting point for our study of fatty infiltration in the musculoskeletal system.

Fatty infiltration is characterized by an abnormal accumulation of adipocytes in non-adipose tissue, where lipid overload produces lipotoxic factors, such as fatty acids and adipokines, resulting in cellular activity, metabolism, and functional abnormalities within musculoskeletal tissues. To explore the mechanisms related to fatty infiltration, starting with the introduction of adipose tissue, this review investigates the effects of fatty infiltration on bone and muscle and finds common causes of this pathological change. Then, from the perspective of the correlation between common clinical musculoskeletal degenerative diseases and fatty infiltration, we will discuss potential future treatments. It is hoped that this will increase public awareness of fatty infiltration of the musculoskeletal system, as well as enrich and improve clinical treatment of degenerative diseases of the musculoskeletal system.

## 2 Adipose tissue

From 1980 until today, adipose tissue, in addition to serving as a structural support for tissues and an inert depot for triacylglycerol storage, has evolved into a dynamic, metabolically active endocrine organ involved in a wide range of biological functions, including glucose and energy homeostasis, immunity, thermogenesis, and insulin resistance (3).

Adipose tissue is categorized into two main groups: brown adipose tissue (BAT) and white adipose tissue (WAT), depending on its localization, composition, and function. BAT is composed of multiple smaller lipid droplets distributed in certain anatomical depots in the neck, posterior thorax, shoulders, and abdomen, with high vascularization and high mitochondrial levels (4). Generally, it does not store energy but functions in adaptive thermogenesis, stimulation of energy expenditure, and regulation of body temperature (5). WAT consists of single lipid droplets located in the viscera and subcutis, which are inferior to BAT in terms of vascularization and innervation. WAT can store excess lipids in the form of triacylglycerol (TAG) and release free fatty acid (FFA) when the organism needs it, participate in multiple organ glucose metabolism, regulate insulin sensitivity, and secretes signaling factors to regulate appetite and energy metabolism homeostasis (6). Interestingly, as the research progressed, a unique type of adipose emerged, although it was not yet well defined. Beige adipose tissue (BeAT) has similar structural features to BAT in that both contain multiple lipid droplets, but its localization is more oriented towards WAT, also called “brown in white” adipocytes. BeAT has the capacity to convert chemical energy into thermogenic energy, which contributes to adaptive thermogenesis and the regulation of energy metabolism (7). Different types of adipose tissue undertake different physiological functions in response to changes in the internal and external environment of the body. When adipose tissue undergoes impaired function and ectopic deposition, it is susceptible to fatty infiltration, resulting in pathologic changes in the organism. In the musculoskeletal system, skeletal muscle fatty infiltration was predominantly BAT, whereas bone marrow fatty infiltration was predominantly bone marrow adipose tissue (MAT, BMAT). And since MAT is located in the specific region of the bone marrow niche, its function and metabolism are also unique from other adipose tissues. (**Table 1**).

**Table 1:** The source, structure, localization, and function of WAT, BAT, BeAT, and MAT.

Types	Source	Structure	Location	Function
**WAT**	Myf5- precursor cell	Monolocular lipid droplets; Low mitochondrial level and vascularization;	Subcutaneous tissue and around the internal organs;	Energy storage as triglycerides; Insulation and immunomodulation; Mechanical support for internal organs; Secretion of adipokines and appetite regulation; Endocrine and energy metabolism regulation;
**BAT**	Myf5+ and Pax7+ progenitor cell	Multiple lipid droplets; High mitochondrial level and vascularization;	In certain anatomical depots in the neck, shoulders, posterior thorax, and abdomen;	Energy dissipation and heat production; Body weight and temperature regulation; Counteracting metabolic diseases; Critical to newborn infants survival;
**BeAT**	Myf5- precursor cell	Multilocular lipid droplets; Related structures still need further study;	Spread throughout WAT	Thermogenic function; Secretion of adipokines; Regulation of energy metabolism;
**MAT**	Progenitor cell expressing osterix	A unilocular lipid droplet; nucleus situated at the cell periphery	Bone marrow niche	Regulation of bone and whole-body energy metabolism; Regulation of bone homeostasis and hematopoiesis;

## 3 Fatty infiltration

Fatty infiltration, an abnormal accumulation of adipocytes in non-adipose tissue, is a process that produces toxicity that has been shown to be involved in a variety of pathological changes, including insulin resistance, renal anemia, heart failure, sarcopenia and osteoporosis (8). Excess dietary lipid supply is often thought to be a major factor in ectopic lipid deposition, but for people on a normal diet, the mismatch between the lipid storage and lipolysis functions of adipose tissue is the key to “fat spillover” (9). When hydrolysis of TAG in tissues exceeds esterification of FFA, a net release of FFA results, which in turn leads to ectopic storage of triglycerides and a cytotoxic reaction (10). Of which, the toxic effects of palmitic acid (PA) and stearic acid in FFA are particularly notable, and most studies in this field also use PA as a lipotoxicity-inducing condition.

In fact, adipose tissue has its own defense mechanism against lipotoxicity, that is, adipose mesenchymal stem cells express differentiation-dependent proteins that bind fatty acids and esterify FFA to neutral triglycerides, thus reducing the toxic response produced by adipocytes (11), but this process can also cause damage to the stem cells themselves, so the defense against lipotoxicity can only be met when the stem cells are in a highly active, metabolic, and value-added state. In other words, stem cells need to be “young and strong.” However, with aging and other pathological factors, the decreased esterification capacity of stem cells leads to the uncontrolled release of adipokines and FFA, a process that not only affects normal metabolism within local tissues, but can also affect the function of neighboring tissues through inter-tissue networks, and even entire systems.

Before proceeding to specific studies of fatty infiltration in the musculoskeletal system, we have to give a preliminary overview of the pathological environment that predisposes to fatty infiltration. First of all, aging, whether it is bone, muscle, or fat, occurs with age-related changes. Aging is a large environment that brings pathological changes not only to the adipocytes themselves, but also to multiple changes such as inflammation, immune decline, and organ hypoplasia, which drive the onset of fatty infiltration in both direct and indirect ways (12). The second is obesity and inflammation, which people tend to equate with increased adiposity. Adipocytes in such individuals undergo hypertrophy, hyperplasia, and activation, leading to the accumulation of pro-inflammatory macrophages and other immune cells, as well as the dysregulated expression of various adipokines (13). In general, the occurrence of fatty infiltration is complex, sometimes the pathological changes it causes are equally responsible for its pathogenesis, often forming a vicious circle.

## 4 Effects of fatty infiltration on bone tissue

### 4.1 Specificity of MAT

MAT originates from progenitors expressing osterix (Sp7) and consists of unilocular lipid droplets, which account for more than 10% of total body fat (14). Unlike osteoblasts and osteoclasts that use glucose metabolism for energy acquisition, MAT relies on FFA released from intracellular lipolysis for oxidative metabolism (15). According to the location of the adipose in the bone marrow, it can be further classified into regulated and constitutive MAT. Regulatory MAT, located in the proximal skeleton, shows active hematopoiesis and bone remodeling, as well as more vulnerability to aging and disease factors. In contrast, the amount of constitutive MAT located in the yellow marrow of the distal skeleton is much more stable and has almost no hematopoietic function (16).

MAT is in contact with hematopoietic cells and bone tissue in the bone marrow niche and is an endocrine-active dynamic fat depot capable of releasing substances such as adipokines, fatty acids, and peptides in paracrine and endocrine forms in response to metabolic demands (17). It has positive effects such as enhancing cortical bone load-bearing capacity, protecting the body from falls and fractures in accidental situations, regulating hematopoiesis, and bone homeostasis, but these beneficial effects will decay due to factors such as aging, anorexia nervosa, and diabetes (18), and sometimes even transform into toxic effects due to excessive proliferation and expansion. Compared to extramedullary fat, MAT is distinctly different in adipogenesis, lipolysis, insulin signaling, inflammation, glucose transporters, and other aspects of immunometabolism (19). However, the specific up-and-down-regulation relationships under different pathologic conditions need further study.

In addition, studies found that when BAT- and WAT-specific gene relative expression analysis was performed on rat tibial bone marrow adipose tissue, MAT was not clearly defined by brown or white adipose tissue, but it was able to express its genes at BAT and WAT specific levels, with a preference for BeAT (20). MAT provides energy support for hematopoiesis and bone metabolism and promotes bone formation when BAT is the characteristic function, while fatty acids, adipokines, and proinflammatory cytokines are mainly secreted when WAT is the characteristic function (6). Therefore, scholars have proposed that the occurrence of intramedullary lipotoxicity may be related to the decreased BAT capacity of MAT and the increased WAT capacity, while both BAT and WAT can regulate energy homeostasis in response to the overall metabolism of the body, and can be transformed into each other under certain conditions. So, it may be a potential way to improve fatty infiltration by regulating the transformation between BAT and WAT. In animal studies, browning of subcutaneous fat has been demonstrated to be induced by exercise in mice, but clinical studies have not been able to reproduce it (21).

In general, the local effects of MAT are mainly in the regulation of bone homeostasis and hematopoiesis, whereas the systemic effects are in immunometabolism. The occurrence of bone marrow fatty infiltration is not only related to localized lesions, but is also influenced by systemic factors.

### 4.2 Marrow fatty infiltration and bone loss

The typical evidence of bone loss in structure is decreased number of bone trabeculae, thinning of the bone cortex, and large infiltration of adipocytes in the bone marrow cavity. With aging and other pathologic factors such as microgravity in spaceflight (22) and prolonged disuse in bedrest (23), the amount of MAT increases and gradually shifts from compensation to derepression, exhibiting more anti-osteogenesis, pro-adipogenic, and pro-apoptotic phenotypes (24). Early on, the increase in MAT is considered more as a supplement to the amount of bone lost in the marrow, but in fact, the numbers of increased adipocytes far exceed the original amount of lost bone, which means that these increased adipocytes have undergone some functional transformation in bone formation.

Marrow fatty infiltration has been observed in mouse genetic models that impair the mechanical loading induced new bone formation. For instance, Pkd1 and/or Wwtr1 deficiency in bone enhances bone marrow fat accumulation and impairs mechanical stimulation induced new bone formation *in vivo* (25, 26). In addition, MAT has powerful endocrine functions that affect postnatal bone homeostasis. It has been found that adipocyte-derived factors can influence the development of key effectors of bone remodeling in a direct or indirect way, thus affecting bone mass and skeletal integrity (18). For example, the release of adipokines and pro-inflammatory factors, such as interleukin 6 (IL-6) and tumor necrosis factor-α (TNF-α), caused by increased MAT expansion is capable of inducing a shift in bone marrow mesenchymal stem cells (BMSCs) from osteogenesis to adipogenesis differentiation (27). The Pa-induced lipotoxic environment also leads to apoptosis and autophagy in BMSCs (28). Disruption of the balance between bone formation and bone resorption by MAT-secreted C1q/tumor necrosis factor (TNF)-related protein-3 (CTRP3), by inhibiting osteoblast-mediated osteoclast differentiation (29). Indeed, endocrine functions of marrow fatty infiltration will impair or deteriorate the original active function of the pre-existing bone marrow microenvironment, leading to abnormal intramedullary bone tissue homeostasis and metabolism (30, 31).

### 4.3 Effect of fatty infiltration on the intramedullary osteogenic lineage

#### 4.3.1 Osteoblast

Osteoblasts and adipocytes both originate from BMSCs (32). The imbalance between osteogenesis and adipogenesis in the bone marrow niche is an important factor contributing to fatty infiltration. Wingless-related integration site (Wnt) and PPARγ are important signaling pathways for osteogenesis and adipogenesis, respectively (33). Wnt signaling reduces the expression and activity of PPARγ and CCAAT/C/EBPα in BMSCs, which inhibits adipocyte formation. Similarly, adipogenesis in BMSCs requires activation of PPARγ signaling and inhibition of osteogenic pathways such as Wnt and Notch (18). This is a complex regulatory network and the relevant cytokines that regulate differentiation are shown in the following table. (**Table 2**).

**Table 2:** Cytokines associated with osteogenesis and adipogenesis in the bone marrow niche.

Cytokine	Function	Source	Ref.
sFRP-1	inhibition of Wnt/β-catenin signaling;	pre-adipocytes	(34)
Legumain	promote adipogenesis; suppress osteoblast commitment and maturation;	adipocytes	(35)
Chemerin	promote adipogenesis; suppressing osteoblastogenesis; activation of CMKLR1 expressed by HSCs promoted osteoclastogenic; differentiation and matrix resorption;	adipocytes	(36)
METRNL	impair osteoblast differentiation and maturation;	adipocytes	(37)
Adenosine	promote both osteoblastogenic and adipogenesis differentiation depending upon the subtype of adenosine receptor activated;	BMSCs	(38)
Adiponectin	Circulating adiponectin levels are associated with lower bone mass;	adipocytes	(39)
DLK1	Inhibition of BMSCs differentiation; negative regulator of OCN;	BMSCs pre-adipocytes	(40)
RARRES2	promote osteoclast differentiation and proliferation; inhibition of Wnt/β-catenin signaling;	adipocytes	(41)
TNF-α	Inhibition of lipoprotein esterase; promotes osteoclast proliferation; Inhibition of osteoblast function;	adipocytes	(42)
IL-6	promotes osteoclastogenesis; enhances osteoclast activity;	adipocytes	(43)

Osteoblasts show markedly impaired differentiation and function under fatty infiltration. High FFAs levels significantly inhibited osteoblast differentiation in human (44). As a natural ligand for PPARγ, FFAs also induce PPARγ activation in BMSCs, preosteoblasts and osteoblasts, and convert osteoblasts into adipocytes by inhibiting Runx2 activity (45). Duque et al. found that lipotoxicity affects the expression of important osteogenic genes such as ALP, Runx2, OCN, OSX, and BSP2, as well as bone mineralization capacity, with a significant decrease in alkaline phosphatase (ALP) and alizarin red staining (46).

Large amounts of osteoblast apoptosis can be observed in the fatty infiltration environment, a process also known as lipoapoptosis. There are two main pathways for apoptosis to occur: an extrinsic pathway that requires transmembrane death receptor mediation, and an intrinsic apoptosis that is initiated through mitochondrial mediation, while osteoblast lipoapoptosis is mediated by both the extrinsic and intrinsic pathways. Reactive oxygen species (ROS) levels were significantly upregulated when osteoblasts were co-cultured with adipocytes. The ROS-activated ERK/P38 signaling pathway contributes to osteoblast apoptosis, which is significantly ameliorated when the pathway is inhibited (47). Furthermore, the low survival rate of osteoblasts with high apoptotic levels, associated with activation of intrinsic apoptotic caspases 3/7. PA-induced human-derived osteoblasts activate the Fas/Jun kinase (JNK) apoptotic pathway and express Bak and Bax, proteins that permeabilize the mitochondrial outer membrane, prompting mitochondria to release cytochrome C into the cytoplasm to trigger apoptosis, and osteoblasts can be rescued from PA-induced lipoapoptosis when the JNK inhibitor SP600125 is added to the culture medium (48).

#### 4.3.2 Osteoclast

An article on new sources of receptor activator for nuclear factor-κB ligand (RANKL) was published in the journal EMB in 2021, demonstrating by single-cell transcriptome sequencing that adipose lineage cells in mice bone marrow are capable of secreting RANKL (49). Therefore, the increase in MAT will inevitably affect RANKL secretion under fatty infiltration.

The secretion of RANKL in bone tissue is mainly done by osteoblasts, osteocytes, proliferative chondrocytes, B cells, and newly discovered intramedullary adipocytes (50). The nuclear factor kappa-B (NF-κB) pathway involved in RANKL is thought to be a key factor in the regulation of osteoclastogenesis and bone metabolism, in which RANKL binds to nuclear factor-kappaB (RANK) on the membrane surface of osteoclasts to drive osteoclast differentiation, cell survival, migration, and attachment (51). Excessive enhancement of bone resorption results in structural damage manifested by decreased bone mass and increased bone fragility. The adipogenic transcription factors C/EBPβ and C/EBPδ were demonstrated to bind to the RANKL promoter and stimulate the transcriptional expression of RANKL, a process that is accompanied by the downregulation of osteoprotegerin (OPG) which blocks the binding of RANKL and RANK (6). However, the mechanism of excessive RANKL production by adipocytes is not well clarified yet, which still needs further research. Inhibition of excessive RANKL secretion by adipocytes may be an important direction for the treatment of osteoporosis. In addition, low-level inflammation caused by the release of cytokines such as monocyte chemotactic protein-1 (MCP-1), also known as C-C motif chemokine ligand 2 (CCL2), tumor necrosis factor-α-associated apoptosis-inducing ligand (TRAIL), IL-6, and TNF-α under fatty infiltration enhances RANKL-induced NF-κB signaling, leading to an exaggerated response and inflammatory osteolysis (52). For instance, TNF-α is able to directly regulate the RANKL-induced signaling pathway, reduce the production of OPG and increase the concentration of RANKL (53). Even some studies have suggested that the presence of PA is sufficient to induce osteoclast differentiation and promote bone resorption regardless of the presence of RANKL in mice (54). So, whether it is the direct stimulation of osteoclasts or the alteration of the bone marrow niche caused by fatty infiltration, there is a promotion of bone resorption, resulting in a disruption of bone homeostasis.

#### 4.3.3 Osteocytes

Fatty infiltration disrupts the normal secretory function of murine long bone osteocytes, and several cytokines, including RANKL, Dickkopf1 (DKK1), and sclerostin, are affected by lipotoxicity. When PA-induced osteocytes were compared with normal culture groups, the fatty infiltration group showed significantly impaired cell viability, lipoapoptosis, and abnormal expression of RANKL, DKK1, and sclerostin (55). Lipotoxicity stimulates osteocytes to secrete sclerostin to activate FAS, a member of the TNF Superfamily Receptors, triggering osteoblasts and osteocytes apoptosis and inhibiting bone morphogenetic protein-induced osteoblastogenesis (56). Dkk1, a negative regulator of bone formation secreted by osteocytes, affects bone mass by inhibiting the Wnt pathway, which has a key role in osteogenesis and bone homeostasis (57). In addition, apoptosis of osteoblasts itself can stimulate osteoclast aggregation and thus enhance bone resorption, further contributing to bone loss.

Interestingly, PA-induced significant increase in lysosomal vital functional protein microtubule-associated protein light chain 3-II (LC3-II) in osteocytes indicated that lysosomal activity was indeed enhanced, but the co-localization of LC3-II and long terminal repeat (LTR) was found to be low after using the lysosomal tracker LTR (55), which means that the enhanced lysosomal activity did not lead to efficacious autophagy. This is at variance with some of the studies, but it is understandable that either the loss of osteocytes due to enhanced autophagy or the inability to clear impaired cells to the accumulation of malignant metabolites due to impaired autophagy can disrupt internal homeostasis and thus affect normal bone metabolism.

**Figure 1:**
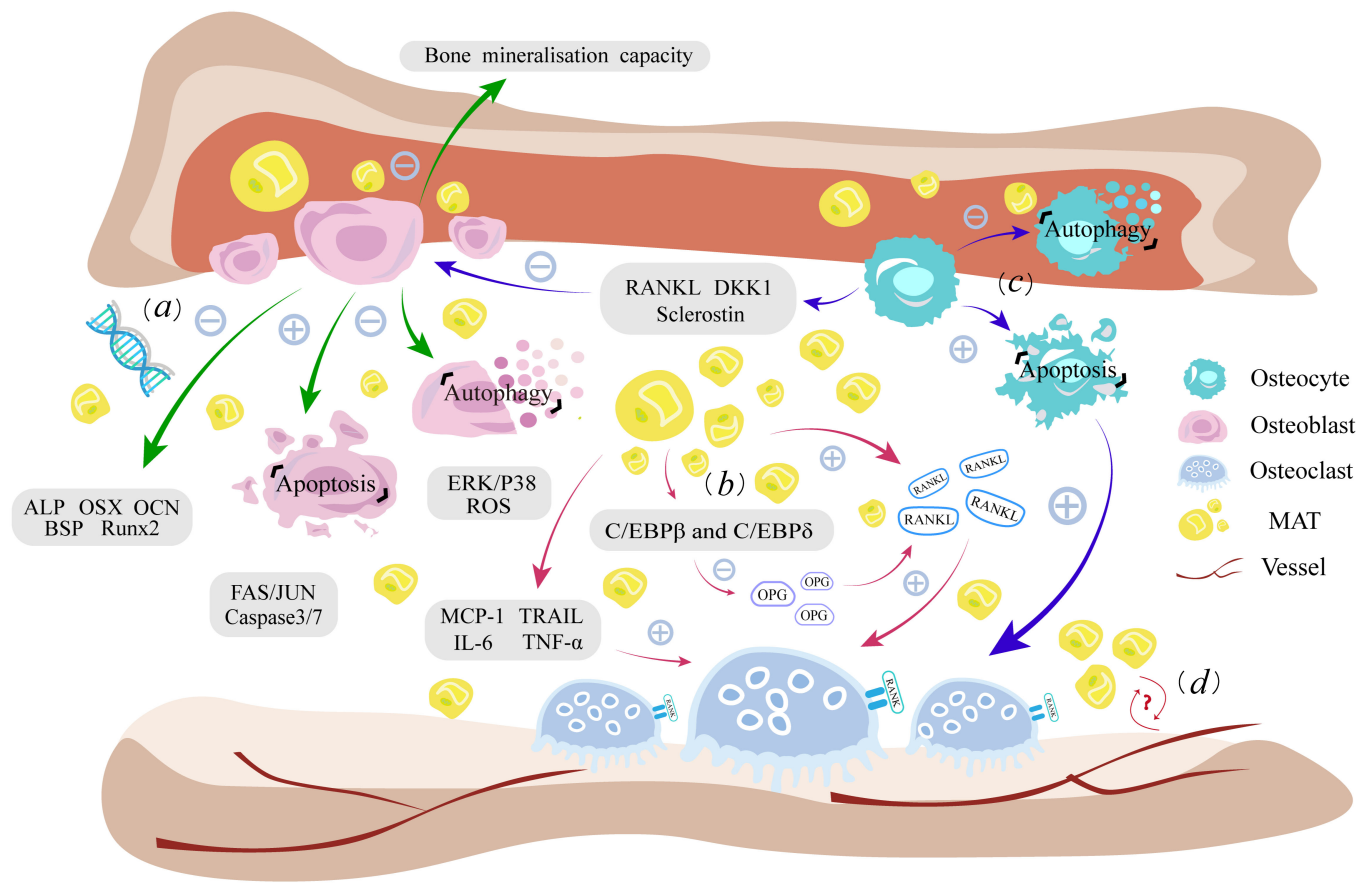
Fatty infiltration damages the homeostasis of intramedullary osteogenic lineage. **(A)**. Lipotoxicity leads to dysfunction of osteoblast autophagy, enhanced lipoapoptosis, weakened bone mineralization, and impaired expression of osteogenic genes. **(B)**. Increased RANKL secretion by fatty infiltration and inflammation caused by adipokines promote osteoclast proliferation, while downregulating the antagonist OPG levels ultimately enhancing the bone resorption process. **(C)**. Osteocytes have a similar lipotoxic response to osteoblasts. Cytokines such as RANKL, DKK1, and sclerostin secreted by osteocytes as well as their own apoptosis, can indirectly affect the function of osteoblasts and osteoclasts. **(D)** MAT is closely related to hematopoiesis and vessels, but the effect of fatty infiltration on it needs further study. RANKL, receptor activator for nuclear factor-κB ligand; RANK, nuclear factor-kappaB; OPG, Osteoprotegerin; MCP-1, monocyte chemotactic protein-1; TRAIL, apoptosis-inducing ligand; IL-6, interleukin 6; TNF-α, tumor necrosis factor-α; ALP, alkaline phosphatase; JNK, Fas/Jun kinase; DKK1, Dickkopf1; MAT, bone marrow adipose tissue; ROS, reactive oxygen species.

In general, the change in the bone marrow niche is closely linked to the raised level of MAT, and the presence of MAT no longer acts as a filler for bone loss, but can secrete relevant factors to influence the overall bone metabolism. Early adipocytes do have some advantageous functions, but with the superimposed effects of aging and various pathological factors, the negative effects of fatty infiltration will severely damage the bone marrow niche, leading to altered differentiation of BMSCs and damage to the above-mentioned intramedullary osteogenic lineage, ultimately resulting in bone loss. (**Figure 1**).

#### 4.3.4 Other impacts

Bone marrow adipocyte lineage cells are capable of expressing dipeptidyl peptidase 4 (DPP4), a protease associated with diabetes treatment (58), which is negatively correlated with bone mass, but no clear mechanism has been proposed (59). MAT also inhibits B-lymphopoiesis by stimulating the production of myeloid-derived suppressor cells and Granulocyte-macrophage colony stimulating factor (GM-CSF) in mice, after which the decrease in OPG production by B cells disrupts OPG/RANKL-mediated bone homeostasis (60).

In addition, numerous studies have found an important relationship between MAT and hematopoietic or blood vessels in the bone marrow. Shafat (61) and Mattiucci et al. (62) found MAT to actively transfer FFA to hematopoietic stem cells (HSC) as well as to secrete CXCL12 *in vitro* cultures, which plays an important role in the mobilization and maintenance of quiescence for HSCs. Zhong et al. (63) discovered a type of marrow adipogenic lineage precursor cell in mouse bone marrow capable of differentiating into adipocytes and increasing with aging. This cell type functions to maintain the marrow vasculature system and to prevent BMSCs from differentiate into osteoblasts. When it was selectively removed from mice, sinusoidal vessels were destroyed and the bone trabeculae were increased (64). Some scholars have suggested that the increase in adipocytes has a negative effect on hematopoiesis by disrupting the original cellular components within the bone marrow niche and intercalating within the hematopoietic milieu, which interferes with the connections between HSCs and other cells (31). Nevertheless, there are a limited number of studies addressing the exploration of the mechanisms of fatty infiltration on hematopoiesis and blood vessels, representing a crucial avenue that warrants further investigation in the future.

## 5 Effect of fatty infiltration on skeletal muscle

Compared with bone tissue, the fatty infiltration of skeletal muscle is more thoroughly studied. Besides a vital organ for exercise, skeletal muscle plays a crucial role in glucose uptake, glycogen storage, lipid oxidation, energy metabolism, and immune function (65). In recent years, it has become increasingly clear that fatty infiltration of skeletal muscle (IMAT, MFI) is an important cause of loss of muscle mass and strength. The increase of adiposity in skeletal muscle not only affects normal muscle metabolism, but also induces a series of pathological changes that cause impairment of skeletal muscle function. Some scholars even suggest that the key indicator for predicting skeletal muscle strength and mobility in the elderly is the degree of skeletal muscle fatty infiltration rather than skeletal muscle mass (66).

Myofibers can be broadly classified as slow- and fast-twitch myofibers, which are often referred to as type I and type II fibers. Type I fibers are typically densely populated with mitochondria and have a high oxidative capacity to support the energy demands of endurance exercise, while type II fibers typically have fewer mitochondria and higher levels of glycolysis for fast bioenergetic metabolism and explosive endurance exercise (67). Myofibers can be further classified into subtypes based on myosin adenosine triphosphatase expression and oxidative metabolism, which have different functional and metabolic properties (68). There are two main forms of fatty infiltration in skeletal muscle, one is intramuscular fat accumulation, which is associated with reduced insulin sensitivity, inflammatory response of the body and impairment of skeletal muscle function. Many skeletal muscle resident cells in the injured status are able to differentiate into intramuscular fat, such as muscle satellite cells (MuSCs), fibro/adipogenic progenitor cells (FAPs), pericytes, myoendothelial cells, fibroblasts, and mesoangioblasts (69); Second is intermuscular fat accumulation, which is associated with impaired function of stem cell populations such as mesenchymal stem cells and FAPs (70). Normally, muscle fat occupies more muscle tissue with aging is a sign of age-dependent muscle tissue damage. However, excess FFA and metabolic intermediates such as diacylglycerol (DAG), long-chain acyl-CoenzymeA, and ceramide produced during lipid metabolism can exacerbate mitochondrial dysfunction, induce insulin resistance and inflammation in skeletal muscle, ultimately affecting fiber homeostasis as well as skeletal muscle generation, apoptosis, and damage repair (65). Interestingly, the researchers also found that the unique structural and functional characteristics of type II fibers make it much more vulnerable to fatty infiltration than type I fibers (71). In the following, we will discuss the skeletal muscle pathological changes caused by fatty infiltration and the specific functional impairment of the muscle fibers.

### 5.1 Pathological changes of fatty infiltration in skeletal muscle

#### 5.1.1 Impaired mitochondrial function

FA accumulated around mitochondria is susceptible to ROS-induced lipid peroxidation and these lipid peroxides may have toxicity effects on mitochondrial mtDNA, RNA, and proteins, leading to mitochondrial dysfunction (72).

ROS-induced endoplasmic reticulum stress is an important cause of skeletal muscle damage. Palmitate treatment in cultured human myotubes induces ER stress markers and decreases cell viability in an *in vitro* study (73). In contrast, oleate treatment (74) or PPARβ/δ activation (75) inhibits ER stress that could ameliorate PA-induced lipotoxicity. Firstly, endoplasmic reticulum stress induces lipolysis and exacerbates fatty infiltration by activating cyclic adenosine monophosphate/protein kinase A (cAMP/PKA) and extracellular signal-related kinase 1 and 2 (ERK1/2) signaling in adipocytes (76). Secondly, the endoplasmic reticulum is unable to provide sufficient protein due to its own impaired function, resulting in a reduced source of myogenesis, and the abnormal modified protein produced during the impairment activates both Caspase3 and fibrillar apoptosis. Thirdly, additional studies have demonstrated that PA-induced endoplasmic reticulum stress will also increase endoplasmic reticulum Ca^2+^ depletion, leading to further skeletal muscle damage from abnormal sphingolipid metabolism and palmitoylation of proteins. In addition, excessive accumulation of ROS in myblast cells activates the NF-κB pathway, leading to an increase in S100B, which induces myoblast transition into brown adipocytes, and negatively regulates miR-133, which has promyogenic and anti-adipogenic effects, through the NF-κB/YY1/miR-133 axis, accompanied by an increase in the brown adipogenic transcription regulator, PRDM-16 (77).

Interestingly, we found that type II fibers in skeletal muscle have stronger oxidative stress. The mitochondrial H2O2 produced/O2 consumed is 2 to 3 times higher in type IIB than normal for basal respiration supported by complex I or complex II substrates. The potential enhanced mitochondrial ROS production capacity of type II fibers (78), coupled with their own special superoxide topology that limits rapid dismutation of O2−•, renders type II fibers more susceptible to oxidative stress than type I fibers (71). This is an important reason for the high sensitivity of tissues to ROS and the poor resistance to lipid stress.

#### 5.1.2 Inflammation

Adipocytes have the function of secreting a variety of cytokines, including pro-inflammatory factors, chemokines, and coagulation factors. Impaired adipose triglyceride lipase (ATGL) and PPARα-mediated lipid signaling pathways were found in skeletal muscle adipocytes in mice with sarcopenia, which resulted in upregulation of pro-inflammatory cytokines such as TNF-α and IL-6 (79). Low-level inflammation caused by fatty infiltration affects redox homeostasis, induces differentiation of adipose mesenchymal stem cells into macrophages and promotes further expression of inflammatory cytokines such as IL-6, TNF-α, IL-1β and MCP-1 (80). Synergy between macrophages and fatty acids also leads to skeletal muscle insulin function impairment (81). The release of certain cytokines enhances the signaling cascade and activates the NF-κB pathway, a pathway whose prolonged activation leads to severe muscle atrophy (82), and reduced insulin sensitivity affecting muscle protein synthesis (83). In addition, TNF-α acts as a strong promoter of TAG hydrolysis in adipocytes, inhibiting the antilipolytic effects of insulin and adenosine by inhibiting insulin receptor signaling and G protein-coupled adenosine receptors signaling, or interacting with the lipid-binding protein perilipin to directly stimulate lipolysis and exacerbate fatty infiltration (65). Impaired activation at the type II neuromuscular junction, excitation-contraction uncoupling, and disruption of the muscle fiber cross-bridge cycle can be observed under inflammation. Fatty infiltration contributes to inflammation, which in turn stimulates the continued accumulation of adipocytes, both of which persist in a vicious cycle and ultimately cause damage to skeletal muscle structure and function.

#### 5.1.3 Insulin resistance

Fatty infiltration, pro-inflammation, oxidative stress, and the accumulation of toxic lipids ceramide and diacylglycerol can induce insulin resistance. While insulin resistance inhibits insulin-mediated antilipolytic capacity, increased plasma levels of FFA provide the precursor conditions for fatty infiltration (76). The release of chemokines such as adipose tissue-activated pattern recognition receptor (PPR) and MCP-1 that recruit macrophages and thus produce pro-inflammatory effects also induces insulin resistance. Insulin sensitivity is strongly negatively correlated with fatty acyl-CoA (FA-CoA) content, and increased FA-CoA due to insulin resistance exacerbates mitochondrial dysfunction via interfering with mitochondrial adenosine triphosphate synthesis through inhibition of the electron transport chain and reduction of the inner mitochondrial membrane potential (84). Increased ROS and local inflammation due to mitochondrial dysfunction leads to activation of JNK, IκB kinase (IKK), protein kinase C (PKC), and p38-mitogen-activated protein kinase (p38-MAPK) stress signaling pathways that inhibit the insulin PI3K/mTOR signaling pathway (85). In addition, the accumulation of lipid metabolites ceramide and diglycerides in skeletal muscle impairs muscle glucose uptake inducing insulin resistance through Phosphoinositide 3-kinase (PI3K) and Insulin Receptor Substrate-1 (IRS-1) signaling by activating protein kinase C (PKCs).

Insulin resistance affects glucose and amino acid transport through the PI3K/Akt/mTOR signaling pathway. PI3K promotes phosphorylation of the protein kinase AKT, and phosphorylated AKT activates TBC1D4 and TBC1D1 to enhance glucose uptake and mediate mTOR activation to affect protein synthesis. Meanwhile, AKT inhibition of Forkhead box O (FOXO) transcription factors activity affects E3 ubiquitin ligase expression, which is responsible for regulating muscular atrophy, and thereby interfers muscle function (82). It was also found that WNT/GSK/β-catenin, a key pathway regulating adipogenesis in FAPs, is stimulated by insulin signaling, and when GSK3 is pharmacologically blocked, it stabilizes β-catenin and inhibits PPARγ expression, thereby blocking adipogenesis in FAPs and limiting steatosis *in vivo* (86). In addition, ceramide and other sphingolipids can promote TNF-α and Caspace3/7-mediated apoptosis (87). In general, fatty infiltration-induced insulin resistance can impair skeletal muscle function in several ways.

### 5.2 Damage to skeletal muscle fibers

#### 5.2.1 Disruption of extracellular matrix remodeling

In the fatty infiltration environment, impaired muscle fiber integrity, excitation-contraction uncoupling, and an imbalance of calcium homeostasis can be observed due to disruption of extracellular matrix remodeling (ECM), thus leading to skeletal muscle dysfunction and atrophy.

Excess FFA caused by fatty infiltration can disrupt myogenic differentiation and myotube formation via the AMPKα/HDAC4/miR-206 pathway in mice (88). Lipotoxicity can also induce the transformation of calcium cyclin isoforms on sarcoplasmic fibers, and excess lipids induce changes in phospholipid composition that affect Sarco/endoplasmic reticulum Ca^2+^-ATPase (SERCA) activity and thus lead to impaired sarcoplasmic reticulum function. In addition, extracellular matrix remodeling and collagen deposition are triggered by lipotoxicity. Extracellular matrix is associated with high fat dietary intake and has an important role in skeletal muscle development, motor endplate function, and glucose metabolism (89), while increased collagen content in skeletal muscle increases the risk of insulin resistance (90). The two pathological changes impair the function of the sarcoplasmic reticulum and also affect the structural integrity of the sarcomeres, fibers, and neuromuscular junctions (71).

Fatty infiltration also affects the damage repair process of muscle fibers, especially MuSCs. Fatty infiltration leads to atrophy and a decrease in the number of MuSCs in type II fibers, suggesting that the stressful environment generated by fatty infiltration can limit the activation, proliferation, and differentiation of MuSCs, thereby diminishing their repair capacity. PAX7, an important marker gene of MuSCs, regulates the myogenic differentiation genes Myod and Myf5, while Myf5 and MyoD are the first two genes expressed in the gene family of the myogenic regulatory factors MRFs. The lack of both Myod and Myf5 results in a complete failure of muscle growth to initiate (91). Another study found that fatty infiltration induced differentiation of mesenchymal stem cells into macrophages, and Interferon-gamma (IFN-γ) secreted by macrophages also had important effects on skeletal muscle damage repair. INF-γ is required in acute muscle injury but is detrimental in chronic injury such as fatty infiltration (92). Increased INF-γ inhibits skeletal muscle damage repair in mice mediated by ERK1/2 and MAPK38 pathways. Moreover, under persistent inflammation, INF-γ impairs the regenerative process by suppressing myogenin expression, but this process can be neutralized by regulatory T cells, providing a potential direction for later therapy (93). In addition, FAPs also affect MuSCs and thus interfere with skeletal muscle damage repair. FAPs help maintain homeostasis within skeletal muscle and assists MuSCs in resolving limited damage in healthy muscle. However, changes in FAPs differentiation tendencies due to the disruption of niche cues can disrupt this balance (94). FAPs themselves have an important role in the development of fatty infiltration which may be the main resident muscle stem cell population involved in the development of ectopic obesity in human skeletal muscle. In a mouse regeneration model, only progenitor cells expressing the cell surface receptor platelet-derived growth factor receptor alpha (PDGFRα) were able to differentiate into adipocytes after glycerol-induced muscle injury, while in the muscle injury state, PDGFRα was expressed only by FAPs, demonstrating the strong lipogenic capacity of FAPs *in vivo* (95). Paracrine factors and soluble molecules secreted by FAPs can regulate the fate of MuSCs. A mouse study found that loss of FAPs-derived WNT1 Induced Signaling Pathway Protein 1 (WISP1) during aging affects the expansion and asymmetric commitment of MuSCs through the Akt signaling pathway (96). Alessio et al. found that inhibition of the key WNT/GSK/β-catenin pathway of FAPs adipogenesis in mice skeletal muscle using an inhibitor was effective in stimulating the differentiation of MuSCs into mature myotubes, thereby improving the pro-myogenic effects of FAPs (97). With a shift of FAPs more towards lipogenesis, the depletion of FAPs can’t maintain muscle mass and support MuSCs proliferation and differentiation during muscle regeneration (98).

#### 5.2.2 Cellular signaling networks involved in skeletal muscle fatty infiltration

Fatty infiltration affects intercellular signaling pathways within skeletal muscle regulating myofiber growth and death. Muscle mass is determined by the balance between protein synthesis and degradation. Skeletal muscle proteolysis is mainly mediated by the ubiquitin-proteasome system (UPS) and the autophagy-lysosomal pathway (ALP) under pathological conditions. Muscle atrophy F-box and MuRF1 are two muscle-specific E3 ubiquitin ligases in UPS system, which are specifically expressed and target specific protein substrates for degradation (99). FOXOs activate both UPS and ALP to ensure that the loss of different cellular components during muscle atrophy is coordinated. Of which, FOXO3 is a key regulator of autophagy as well as controls the expression of other autophagy-related genes (100). Protein synthesis is mainly regulated by the Insulin-like growth factor-1 (IGF-1)/PI3K/Akt/mTOR signaling axis (101). mTOR is an essential factor mediating skeletal muscle growth, the activation of which inhibits UPS and ALP. Nutrients, mechanical and neural signals activate mTOR, while inflammatory cytokines and unfolded proteins inhibit mTOR activity (102). Insulin/IGF-1, which are upstream of mTOR, indirectly regulate muscle growth. Under fatty infiltration, many key inflammatory mediators are involved in skeletal muscle atrophy. High levels of lipid metabolites such as TNF-α, IL-6 and IGF-1 activate MuRF-1 and MAFBx/Atrogin-1, key transcription factors of muscle atrophy, through the IGF/Akt-1 pathway. In additional, TNF-α regulates apoptosis by inhibiting the Akt/mTOR pathway (103). Its lead to prolonged activation of the NF-κB pathway can also lead to severe muscle atrophy.

Additional pathways involved in fatty infiltration are AMP-activated protein kinases (AMPKs), mitogen-activated protein kinases (MAPKs) and histone deacetylases (HDACs), etc. AMPK is a positive regulator involved in glucose transport, mitochondrial function, and fatty acid oxidation, as well as a negative regulator of inflammation, oxidative stress, ceramide and DAG production, and mTOR-related pathways. MAPKs are serine/threonine-protein kinases, and recent studies have demonstrated that the MAPKs family has a central role in the regulation of adipose tissue function. ERK1/2, JNKs, and p38s, which are involved in endoplasmic reticulum stress and insulin resistance mentioned above, are all important members of this family (104). Moreover, HDACs mediated FOXO nuclear localization and activation and enhanced FOXO activity and function through deacetylation, while HDACs regulated the expression of the Atrogin-1 (105). Fatty infiltration involves the activation of multiple signaling pathways within skeletal muscle, affecting skeletal muscle growth, lipolysis, and regulating the expression of genes associated with skeletal muscle atrophy.

**Figure 2:**
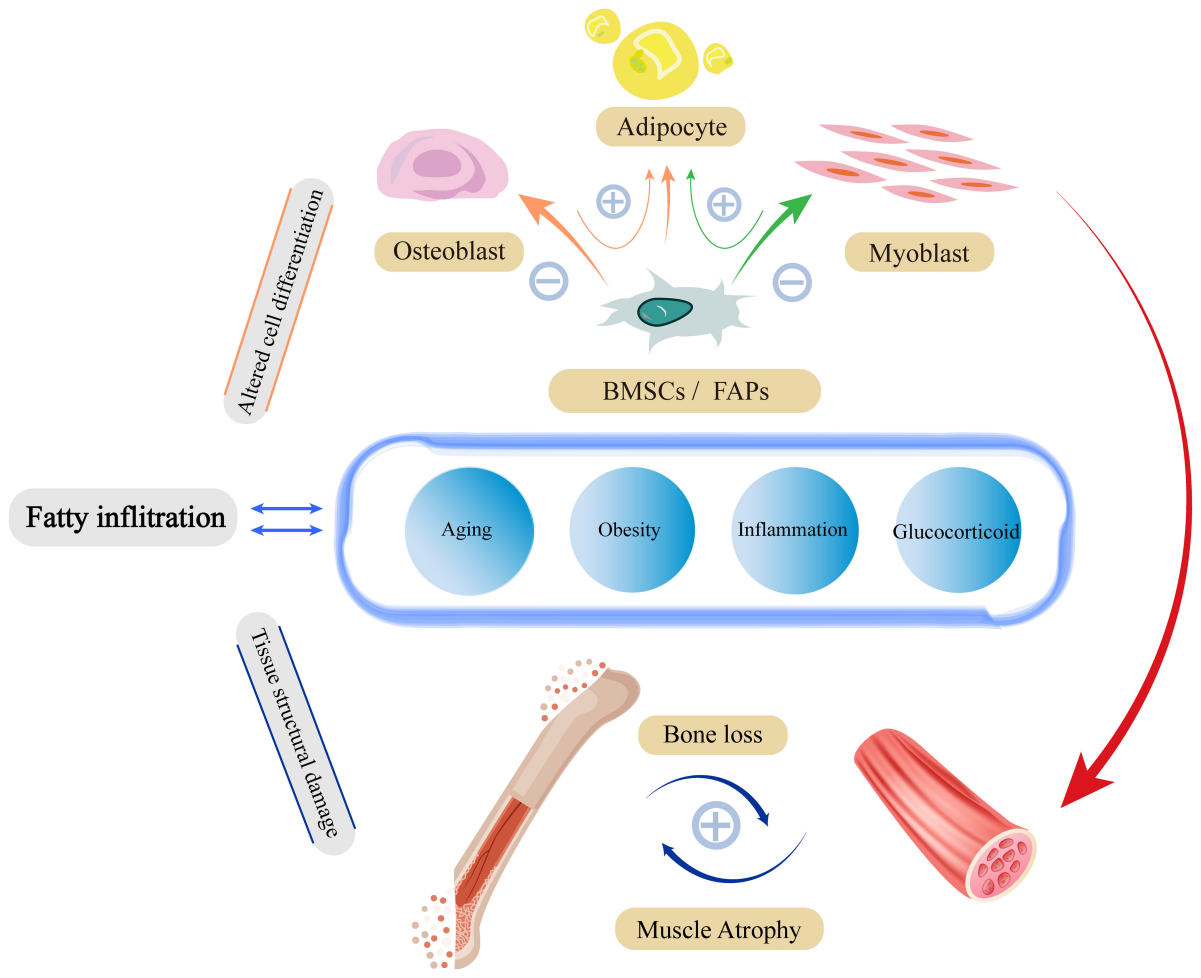
Fatty infiltration damage to skeletal muscle structure and function. Damage to skeletal muscle by fatty infiltration is mainly caused by three pathological alterations through insulin resistance, inflammation, and mitochondrial damage. These pathological states have malignant effects on fibrin-homeostasis, myogenesis, and damage repair functions through complex signaling pathways. To a certain extent, pathological alterations due to fatty infiltration will in turn stimulate further fatty infiltration, forming a vicious circle.

Lipotoxicity impairs mitochondrial function, increases ROS release, persistent low level chronic inflammation and insulin resistance will disrupt fibrin homeostasis and affect skeletal muscle generation, apoptosis and damage repair, and these major functional lesions will in turn further stimulate lipid accumulation, creating a vicious cycle. The structural and functional specificity of type II fibers is such that they differ markedly from type I fibers in their level of response to fatty infiltration (**Figure 2**).

## 6 Extrinsic factors causing fatty infiltration

**Figure 3:**
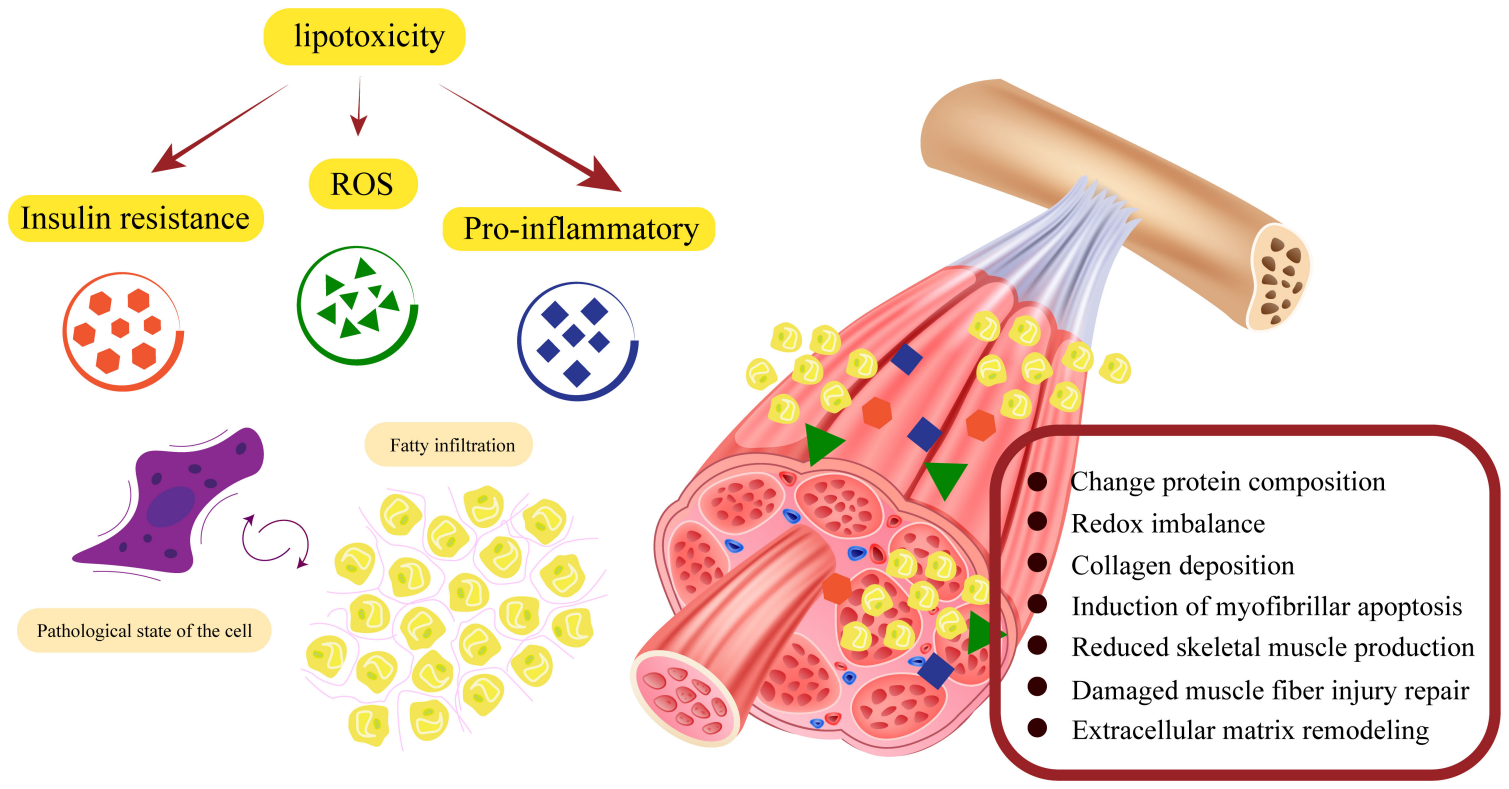
Factors affecting the fatty infiltration of the musculoskeletal system. Aging, insulin resistance, oxidative stress, and inflammation are important factors that exacerbate fatty infiltration. 1. Mesenchymal stem cells have the ability to differentiate into osteoblasts, myoblasts and adipocytes. Fatty infiltration impairs osteogenic and myogenic differentiation of stem cells. At the same time, lipotoxicity promotes the shift of stem cells from osteogenesis and myogenesis to lipogenesis, ultimately leading to an overall disruption of musculoskeletal metabolism. 2. The complex network of interactions between muscle and bone makes it possible for damage to one side to often lead to simultaneous damage to the other, eventually leading to structural damage to the musculoskeleton.

Bone and muscle are mechanically stressed tissues derived from mesodermal cells that show significant degenerative changes in mass and strength with age (70). Their damage and degeneration are reciprocal, not alone, and are influenced by the interaction of mechanical stress stimuli and endocrine networks. In turn, fat is an important player in this “crosstalk” (106) (**Figure 3**).

### 6.1 Aging

Aging is a topic that cannot be avoided when discussing fatty infiltration and the musculoskeletal system, and excessive accumulation of adipose in non-adipose tissues such as the musculoskeletal is regarded as a feature of aging. First, aging causes a decrease in the ability of adipose mesenchymal stem cells to mitigate their own lipotoxicity, and induces a shift in stem cell differentiation from osteogenic and myogenic to lipogenic, which directly affects musculoskeletal metabolism. Second, aging changes the secretion level of other cytokines to indirectly induce the formation of fatty infiltration.

Both hypertrophic and hyperplasia forms of adipocyte pathological alterations, can be observed in senescent tissue. Morphological changes in senescent adipose tissue impair metabolic activity leading to insulin resistance, inflammation, and energy expenditure reduction (12). Although cell enlargement allows adipose tissue to store more lipids, thus mitigating to some extent the effects of lipotoxicity in other organs, the decrease in surface-to-volume ratio due to hypertrophy results in a decrease in the efficiency of cell signaling and nutrient transport (107). Simultaneously, the internal environment of adipose tissue undergoes significant changes, such as reduction of mitochondria, enlargement of lipid droplets, and infiltration of inflammatory factors and immune cells, which will lead to pathological changes, like insulin resistance and inflammation (108).

Bone and muscle both show age-related degeneration. Studies have demonstrated that bone loss with aging is exacerbated by the downregulation of osteogenic genes such as Runx2 and OCN and the upregulation of PPAR-γ and adipocyte fatty acid-binding protein (aP2), also known as Fatty Acid Binding Protein 4 (FABP4). Similarly, genes related to skeletal muscle cell differentiation, tissue fibrosis, angiogenesis, and mitochondrial lipid regulation are also downregulated with aging (109). In addition, aging leads to changes in hormone levels, insulin resistance, inflammation and other pathological factors that can also affect musculoskeletal metabolism. The silent information regulator sirtuin 1 (SirT1) is expressed in musculoskeletons and decreases with aging (110), SirT1 deficiency increases adipocyte and osteoclast formation, enhances inflammatory responses, and affects musculoskeletal metabolism via NF-κB and PPAR-γ pathways (111). Estrogen, a key regulator of lipid metabolism, also declines with age, and studies have shown that postmenopausal women lacking estrogen stimulation are more likely to suffer from osteoporosis (112). Beekman et al. found that OVX not only increased RANKL production in MAT but also induced the expansion of MAT volume in C3H/HeJ mice (113). Estrogen also reduces muscle injury caused by inflammation, inhibits energy dissipation to maintain efficient mitochondrial energy production and stimulates the activation and proliferation of satellite cells through the estrogen receptor ERα/β to promote muscle damage repair (114). Moreover, mechanical unloading, glucocorticoid use, FOXO, IGF-1, and mTOR also change with aging and affect fatty infiltration (115).

### 6.2 Obesity and inflammation

From the earliest single osteoporosis, to co-morbid osteosarcopenia, and nowadays osteosarcopenia obesity, growing evidence suggests a shared mechanism between excessive adiposity and decreased bone mass and skeletal muscle mass (116). Obesity has a positive effect on bone density in terms of mechanical loading, but this is not sufficient. Metabolic changes can obliterate the positive effects of stress stimulation. As stem cells age, or under the influence of obesity and low-level inflammation, the infiltration of adipocytes into muscle and bone becomes evident, leading to the replacement of muscle and bone cells by adipocytes. As for the skeletal muscle, excessive obesity and coexisting low skeletal muscle mass and/or function have been defined as sarcopenic obesity, a clinically common disease (117).

Patients with obesity exhibit significant metabolic abnormalities: including up-regulation of estrogen, leptin, resistin, TNF-α and IL-6 levels and down-regulation of adiponectin levels (118). Alterations in these hormones and cytokines have important effects on the musculoskeletal system. For example, TNF-α and IL-6 have been demonstrated to affect musculoskeletal metabolism by disrupting osteoblast-osteoclast homeostasis, inhibiting myogenic cell differentiation and proliferation, and accelerating skeletal muscle degradation (106). The effects of chronic inflammation on the musculoskeletal system are summarized as follows (**Table 3**). Interestingly, it was found that although the increase in intramyocellular lipid led to abnormal metabolism of muscle fibers, no significant difference in peak active tension or passive viscoelasticity was observed between the metabolically abnormal group and healthy controls in mice and people, meaning that the IMCL parameter did not have a significant relationship with biophysical variables (126). Certainly, this needs to be explored and validated by subsequent studies over time. Moreover, in the study by Xabier (127), it was found that adipocytes secreted WNT-5A, lipocalin-2 (LCN-2), tenascin C (TNC), calprotectin, and interleukin-32 (IL-32), which also play a key role in inflammation. Obesity and its induced inflammation can induce the development of fatty infiltration, while adipokines secreted by fatty infiltration can exacerbate systemic low-level inflammation, forming a vicious cycle that exacerbates the pathological tendency of BMSCs toward adipogenesis.

**Table 3:** The role of inflammatory factors under the musculoskeletal system.

Adipokines	Function	Source	Ref.
TNF-α↑	Inhibition of lipoprotein esterase and regulation of fatty acid uptake; Reduction of GLUT4 expression in adipocytes and skeletal muscle; Promotes osteoclast proliferation but inhibits apoptosis; Inhibition of osteoblast function and induction of insulin resistance;	Adipocytes; Macrophages;	(42)
IL-6 ↑	Stimulation of RANKL expression promotes osteoclastogenesis; Up-regulation of prostaglandin E2 and IL-1 enhances osteoclast activity; Enhance lipolysis; Activation of skeletal muscle oxidative metabolism;	Osteoblast; Osteocyte; Macrophages; Neutrophils; T cells;	(119)
FGF-21 ↑	Reduction of osteoblast production via PPAR pathway; Stimulation of adipogenesis in bone marrow and inhibition of bone formation; Impacts glucose and lipid catabolism, fatty acid oxidation, and mitochondrial oxidative activity; Induces systemic inflammation and a reduction in myogenesis;	Adipocytes;	(120)
Resistin ↑	Reduces insulin sensitivity and promotes inflammation; Down-regulates glucose tolerance;	TNF-α; IL-6; Macrophages;	(121)
IL-32 ↑	Extracellular matrix remodeling; Stimulation of IL-8, IL-6, IL-1α, and TNF-α secretion; Induction of insulin resistance;	Nk cell; monocytes; Macrophages; T-lymphocytes; Fibroblasts;	(122)
Leptin↑	Increase the production of TNF and IL-6 by monocytes and stimulate the production of CC-chemokine ligands; Regulate energy homeostasis, hypothalamic-pituitary function; Influence levels of thyroid hormones, growth hormones, cortisol, and sex steroids; (There is still disagreement about whether the effect of leptin on bone metabolism is beneficial or harmful);	Adipocytes	(123)
SFRP5↓	Impairment of insulin sensitivity; Exacerbation of metabolic dysfunction and enhanced production of pro-inflammatory cytokines; Increased accumulation of macrophages Enhancement of pro-inflammatory WNT signaling	Adipocytes; Endothelial cells; Muscle cells;	(124)
Adiponectin↓	Reduce insulin sensitivity; Pro-inflammatory and promotion of TNF production; Enhancement of macrophage-mediated inflammation; Exacerbation of glucose intolerance;	Adipocytes	(125)

“↑” indicates that fatty infiltration leads to upregulation of inflammatory factor expression. “↓” indicates that fatty infiltration leads to downregulation of inflammatory factor expression.

In addition, obesity increases the risk of insulin resistance and type 2 diabetes (128). Diabetic bone disease patients exhibit variable degrees of bone loss and increased bone fragility with continued diabetes mellitus, which increases the risk of fracture and impairs the ability to heal after a fracture (129). Hyperglycemia due to insulin resistance has a toxicity effect on BMSCs differentiation, and high glucose levels and its induced advanced glycation end products (AGEs) increase osteocytes’ expression of sclerostin, while stimulating the noncanonical Wnt/PKC pathway and upregulating PPARγ, leading to increased adipogenesis and bone loss (130). The relationship between insulin resistance and muscle fat infiltration has been described in detail in the skeletal muscle part, and will not be repeated here. Overall, obesity, inflammation, insulin resistance, and diabetes form a complex network of attacks on the musculoskeletal system.

### 6.3 Glucocorticoid

Glucocorticoids have an important role in anti-allergic, immune-suppressive, and anti-inflammatory aspects (131). Although glucocorticoids have been criticized for their own side effects, the efficacy in diseases such as asthma (132), systemic lupus erythematosus (133) and vasculitis (134) is certain. In cases of unavoidable glucocorticoid use, we can’t ignore the damage to the musculoskeletal system.

Maier et al. (135) found a significant increase in lipid droplet size and proportional area consisting of lipids in dexamethasone-treated 3T3-L1 adipocytes. Low doses or short exposure times to glucocorticoids tended to promote lipolysis, whereas high doses and long exposure times would result in increased lipid deposition in 3T3-L1 adipocytes. In conclusion, glucocorticoids, due to own characteristics, inevitably pose bone loss and muscle atrophy risks when used. Bone marrow adiposity accumulation is often accompanied by reductions in bone mineral density and trabecular bone in the long bones of glucocorticoid-treated animals, even preceding these concomitant signs (136). In another similar study, glucocorticoid treatment induced a lipid metabolism circuit. Glucocorticoids increased the synthesis of oxylipins, activated PPARγ, and triggered the senescence of bone-marrow adipocyte lineage cells, which in turn acted as mediators of glucocorticoid-induced bone deterioration (137). In addition, it is well established that glucocorticoids induce skeletal muscle atrophy. GC-induced muscle atrophy is caused by increased protein breakdown and decreased protein synthesis. In particular, the activation of the ubiquitin proteasome and lysosomal system in the catabolic action of GC (138). Therefore, it is important to regulate the use of glucocorticoids and add other treatments to ameliorate their side effects.

## 7 Fatty infiltration and diseases of the musculoskeletal system

### 7.1 Osteoporosis and fatty infiltration

Osteoporosis is a systemic bone disease characterized by reduced bone mass, impaired bone quality, and reduced bone strength, resulting in increased bone fragility and susceptibility to fracture (139). The 2019 American Geriatrics Society and National Institute on Aging research conference highlights the relationship between osteoporosis occurrence and soft tissue (muscle and fat) disorders (140). In both animal experiments and clinical studies, MAT ultimately dominates the majority of the bone marrow microenvironment in osteoporotic bones. The negative correlation between low bone mass and increased MAT is evidence that fatty infiltration is indeed an important marker of osteoporosis. Higher MAT and lower bone mineral density (BMD) had a higher rate of cone fractures, while changes in proton density fat fraction (MRI-PDFF) and lipid composition were associated with an increased risk of fracture (141). Moreover, as common lipid-lowering drugs, statins have a positive effect on improving overall rate of fracture and enhancing bone mineral density in the treatment of osteoporosis (142). Simvastatin inhibited the gene expression of lipoprotein lipase and PPAR γ 2 in rats (143). However, it is worth noting that statins have a significant dose-dependent in the treatment of osteoporosis (144). Although there is no clear theoretical basis, it is possible that the modulation of osteoblasts and osteoclasts by statins is achieved through the inhibition of fatty infiltration. In addition, PTH and risedronate, which are now commonly used in osteoporosis clinics, have also been found to have an inhibitory effect on intramedullary adiposity while regulating bone homeostasis (145). In general, combined with studies of lipotoxicity on intramedullary osteogenic lineage, these clinical studies demonstrate that fatty infiltration is an important factor causing osteoporosis and that adipose is the key to treating osteoporosis. So, it is therefore practical and imperative to devise an appropriate treatment plan for the fatty infiltration of the patient.

### 7.2 Sarcopenia and fatty infiltration

Sarcopenia is a syndrome characterized by progressive and generalized loss of skeletal muscle mass and strength (146), and is affected by multiple factors such as insulin resistance, chronic inflammation, ROS (147). As research progresses, the role of fatty infiltration in sarcopenia has become more prominent, with strong associations with mitochondrial damage, pro-inflammatory factor release, and insulin resistance. Moreover, the impairment of muscle structure and function by skeletal muscle fatty infiltration is more obvious in aging and pathological states (148). Obesity exacerbates the development of sarcopenia, increases fat penetration into muscle, reduces physical function, and increases the risk of death (13). In addition, the main pathological change in sarcopenia is the atrophy and reduction of type II fibers. The special function and structure of type II fibers make them more susceptible to fatty infiltration compared to type I fibers, which also verifies the importance of adiposity in muscle pathological changes. This is why clinical studies of muscle fat imaging are increasingly valued, and some researchers regard it as a major criterion for disease diagnosis.

Based on the above studies combined with clinical examples, the relationship between fat, muscle, and bone speaks for itself, accompanied by the cross-linking of obesity, osteoporosis, and sarcopenia. Osteoporosis and sarcopenia are the most typical and prevalent diseases within the musculoskeletal system. In contrast, osteosarcopenia and osteosarcopenic obesity are novel research directions based on the close association between the three. Along with conventional treatment, the application of modern imaging techniques that focus on the effects of fatty infiltration on the disease and inhibit further infiltration of adiposity into the tissues is an important tool in the potential treatment of diseases of the musculoskeletal system.

## 8 Potential therapeutic directions

Based on the occurrence, development, and toxicity effects of fatty infiltration, we summarize the potential treatment into three aspects. First, inhibiting the ability of adipose tissue to produce toxic substances or restoring and improving the defense mechanisms of adipose tissue against lipotoxicity; Second, promoting autophagy of toxic substances and inhibiting lipoapoptosis; Third, shifting the cell differentiation pathway from lipogenesis to osteogenesis and myogenesis. Nevertheless, there are no definitive clinical treatment options for fatty infiltration of the musculoskeletal system, and only partial studies have been found in the skeletal and muscular one-sided fields.

Induction of adipose browning may be an effective way to inhibit lipotoxicity. In this regard, the bidirectional effects of IL-6 are amazing. Low-level inflammation involving IL-6 is detrimental to both the bone marrow microenvironment and skeletal muscle metabolism. However, it was found that human deep neck tissue biopsies rich in BAT released significantly higher levels of IL-6 than subcutaneous biopsies. During beige differentiation, continuous blockade of IL-6 receptors by specific antibodies leads to downregulation of BAT marker genes and differentiation to WAT (149). This indicates that IL-6 has a positive effect on adipose browning, but whether this positive effect can be applied to the bone marrow niche remains to be demonstrated. In addition, unlike chronic inflammation, exercise-induced low or transient IL-6 elevation is beneficial to muscle fiber growth and muscle mass, and even has some anti-inflammatory effects (150).

The above-mentioned mTOR associated with senescence, the study found that rapamycin-induced inhibition of mTORC1 was able to maintain osteoblast viability and reduce PA-induced lipoapoptosis (151).Further, liver X receptor activation increased stearoyl coenzyme A 9-desaturase 1 expression significantly reduced cell death, caspase 3/7 activation, endoplasmic reticulum stress and inflammation caused by lipotoxicity in huamn (152). Meanwhile, LXR possesses the capability to reduce adipocytes, osteoclast differentiation, promote osteoblastogenesis, restore insulin sensitivity, and reduce the secretion of the pro-inflammatory cytokine IL-6. However, its treatment produces side effects that exacerbate steatosis and require treatment in conjunction with other drugs (153). FABP4 is mainly expressed by macrophages and adipose tissue, regulating fatty acid storage and lipolysis, as well as being an important mediator of inflammation. PA can mediate macrophage apoptosis via upregulation of FABP4, which in turn leads to mitochondrial dysfunction and ROS, a pathway that can be inhibited by the FABP4 inhibitor BMS309403 (154). FABP4 inhibitors have similarly positive effects on insulin resistance, inflammation, diabetes, and other metabolic syndromes, but these studies have not been trialed in humans (155). Based on MRI and related analyses, Mansour found that long-term high-intensity endurance training was effective in preventing further development of thigh muscle fatty infiltration in elderly men with early stage sarcopenia (156). In addition, statins, N-acetylcysteine (157), vitamin D (158), resveratrol (159), increasing sarcolipin expression (160) and resistance training also have a role in the treatment of fatty infiltration. The efficacy of these drugs in clinical trials and the optimal utilization to maximize effectiveness while minimizing side effects are still under investigation. In fact, the key to fatty infiltration therapy lies in how to inhibit the lipogenic differentiation of stem cells, or even directly reverse lipogenesis to inhibit the production of lipotoxicity from the origin, but most of the current studies focus on the removal of toxic substances and alleviate the damage caused by lipotoxicity to surrounding cells and tissues, how to regulate the differentiation of stem cells is still a major difficulty in this field.

## 9 Conclusion

With the advancement of modern technology, research on fatty infiltration has become more comprehensive within the musculoskeletal system. The shared precursor cells among muscle, bone, and fat inevitably establish an intricate connection among the three. In addition to impacting individual conditions like osteosarcopenia, novel diseases such as osteosarcopenic obesity have also emerged as derivatives of fatty infiltration. Lipotoxicity inhibits osteocytogenesis and osteoblastogenesis while enhancing their lipoapoptosis, and enhances osteoclast-mediated bone resorption, triggering bone loss. In addition, myofibrogenesis, apoptosis, and damage repair are also affected by fatty infiltration, which ultimately leads to total structural and functional impairment of skeletal muscle. We conclude that aging, obesity, inflammation, and glucocorticoid use have an essential role in the effects of fatty infiltration on the musculoskeletal system, which is also determined by the close connection between musculoskeletal function and structure. At last, we summarized some potential directions for fatty infiltration treatment and related drugs, but how to construct a stable musculoskeletal fatty infiltration model, whether tendon, ligament, and cartilage components of the system are also affected by lipotoxicity, whether there is a difference in the toxicity effect produced by different localization of adipose in the same tissue, how to quantify fatty infiltration by imaging for clinical diagnosis and the related grading of fatty infiltration still need to be further explored. It is hoped that this review will provide researchers with a clearer understanding of the impact of fatty infiltration on diseases of the musculoskeletal system and provide new directions for the clinical diagnosis of similar diseases.

## Author contributions

YHZ: Writing – original draft, Writing – review & editing. YH: Writing – original draft, Writing – review & editing. YP: Writing – original draft. MZL: Writing – original draft. YN: Writing – original draft. TZ: Writing – original draft. HS: Writing – original draft. SZ: Writing – original draft. MML: Writing – original draft. YZ: Writing – original draft. CW: Writing – original draft. YM: Writing – original draft, Writing – review & editing. YG: Writing – original draft, Writing – review & editing. LW: Writing – original draft, Writing – review & editing.
